# Wnt/β-Catenin–mTOR–autophagy crosstalk in breast cancer: context-dependent control of tumor progression, immune suppression, and therapeutic resistance

**DOI:** 10.3389/fimmu.2026.1842528

**Published:** 2026-06-30

**Authors:** Jian Zhang, Chuqin Liu, Yunjian Zhang

**Affiliations:** 1 Department of General Surgery, GuangZhou Eighth People’s Hospital, Guang Zhou Medical University, Guangzhou, China; 2 Department of Breast Surgery, the First Affiliated Hospital of Sun Yat-sen University, Guangzhou, China

**Keywords:** autophagy flux, breast cancer, mTOR, myeloid-derived suppressor cells, Wnt/β-catenin signaling

## Abstract

Autophagy plays paradoxical roles in breast cancer, functioning as both a stress-adaptive survival mechanism and a tumor-suppressive process depending on cellular and microenvironmental context. However, the molecular logic governing this functional duality has remained incompletely understood. Emerging evidence identifies the Wnt/β-catenin–mTOR axis as a central signaling hub that integrates proliferative, metabolic, and developmental cues to determine the directionality and functional outcome of autophagy. Aberrant activation of Wnt/β-catenin signaling reinforces mTOR activity, suppresses autophagic flux, and promotes stemness, epithelial–mesenchymal transition, therapeutic resistance, and immune evasion. Conversely, inhibition of Wnt signaling can relieve mTOR-mediated autophagy repression, leading to context-dependent induction of cytotoxic or cytostatic autophagy. Beyond tumor cell–intrinsic effects, Wnt–mTOR–autophagy crosstalk critically shapes the tumor immune microenvironment. In particular, SOCS3 deficiency–driven activation of Wnt/mTOR signaling represses autophagy in early-stage myeloid-derived suppressor cells, thereby sustaining their survival and immunosuppressive function. This mechanism highlights autophagy as an immunometabolic fate switch that governs myeloid cell persistence and antitumor immune suppression. Importantly, autophagy also feeds back to restrain Wnt signaling through selective degradation of pathway components, establishing dynamic regulatory loops that fine-tune oncogenic signaling output. In this review, we synthesize current evidence to delineate the bidirectional crosstalk between Wnt/β-catenin signaling, mTOR activity, and autophagy in breast cancer. We discuss how this integrated network governs tumor cell states, immune suppression, and therapeutic responsiveness, and propose a biomarker-driven, context-specific framework for autophagy modulation. By integrating signaling, autophagy flux, and myeloid immune status, precision targeting of the Wnt–mTOR–autophagy axis may offer new opportunities to overcome therapeutic resistance and improve clinical outcomes in breast cancer.

## Highlights

- Wnt/β-catenin–mTOR signaling acts as a central gatekeeper that determines the functional orientation of autophagy in breast cancer.
- Autophagy functions as an immunometabolic fate switch in the tumor immune microenvironment, particularly in SOCS3-deficient myeloid-derived suppressor cells.
- Context-specific, biomarker-guided modulation of autophagy is essential for effective combination therapies targeting tumor cells and immune suppression.

## 1 Introduction

Breast cancer remains one of the most heterogeneous malignancies, encompassing distinct molecular subtypes with divergent biological behaviors, therapeutic vulnerabilities, and clinical outcomes (1–3). Among them, triple-negative breast cancer (TNBC) is characterized by aggressive growth, pronounced intratumoral heterogeneity, enrichment of stem-like cell populations, and a high propensity for therapeutic resistance and relapse (4). Increasing evidence indicates that these malignant traits are not solely determined by tumor cell–intrinsic genetic alterations but are profoundly shaped by dynamic signaling networks that integrate proliferative cues, metabolic states, stress-adaptive programs, and the tumor immune microenvironment (TIME).

Autophagy, an evolutionarily conserved lysosomal degradation pathway, has emerged as a central stress-adaptive mechanism in breast cancer (5–9). By recycling intracellular components, autophagy enables tumor cells to survive nutrient deprivation, hypoxia, oxidative stress, and therapeutic insults. In this context, autophagy frequently functions as a cytoprotective program, sustaining tumor cell viability, promoting cancer stem cell (CSC) maintenance, and facilitating resistance to chemotherapy, endocrine therapy, and targeted agents (10–13). Conversely, under specific genetic, metabolic, or pharmacological conditions, excessive or dysregulated autophagy can drive autophagic cell death or cooperate with apoptosis to suppress tumor growth. This functional duality positions autophagy as a prototypical “double-edged sword” in breast cancer biology, raising a critical translational question: when should autophagy be therapeutically induced, and when should it be inhibited?

Resolving this paradox requires an integrated understanding of the upstream signaling pathways that determine the directionality and functional outcome of autophagy. Among these, the Wnt/β-catenin and mammalian target of rapamycin (mTOR) pathways represent two highly conserved and interconnected signaling hubs that orchestrate cell proliferation, metabolic reprogramming, stemness, and stress responses (14–18). Canonical Wnt/β-catenin signaling is a key driver of breast cancer progression, particularly in TNBC and stem-like tumor cell subsets, where it promotes self-renewal, epithelial–mesenchymal transition (EMT), and immune evasion. mTOR, as a master regulator of cellular anabolism and energy sensing, directly controls autophagy initiation through the ULK1 complex and governs lysosomal biogenesis and autophagic flux via transcriptional regulators such as TFEB (19–21).

Importantly, accumulating evidence indicates that Wnt/β-catenin and mTOR signaling do not operate as linear, independent pathways but instead converge to form a regulatory axis that sets the threshold, magnitude, and functional consequences of autophagy (22–25). Activation of Wnt/β-catenin signaling can reinforce mTOR activity, thereby suppressing autophagy and favoring tumor cell survival, stemness, and therapy resistance. Conversely, pharmacological or genetic inhibition of Wnt signaling may relieve mTOR-mediated autophagy repression, leading to autophagy induction with context-dependent antitumor or protumor effects. Adding further complexity, autophagy itself can reciprocally modulate Wnt signaling by selectively degrading pathway components, establishing feedback loops that fine-tune signaling output and cellular fate.

Beyond tumor cells, the Wnt–mTOR–autophagy axis is increasingly recognized as a determinant of immune cell function within the TIME. Autophagy critically regulates the survival, differentiation, and suppressive activity of myeloid-derived suppressor cells (MDSCs), macrophages, and other immune populations, thereby shaping antitumor immunity and therapeutic responsiveness (26–30). Dysregulation of Wnt/mTOR-controlled autophagy in these compartments may promote immune evasion and limit the efficacy of both conventional therapies and emerging immunotherapies.

In this review, we synthesize current evidence to delineate the molecular crosstalk between Wnt/β-catenin signaling, mTOR activity, and autophagy in breast cancer. We focus on how this interconnected network governs tumor cell fate, stemness, therapeutic resistance, and myeloid-driven immunosuppression in a context-dependent manner. By framing autophagy as a signaling-integrated decision node rather than a unidirectional pathway, we aim to provide a conceptual framework for rational therapeutic strategies that selectively induce or inhibit autophagy according to tumor and microenvironmental states.

## 2 Core wiring: how Wnt/β-catenin signaling sets the autophagy switch through mTOR

### 2.1 Canonical Wnt/β-catenin pathway essentials

**Figure 1:**
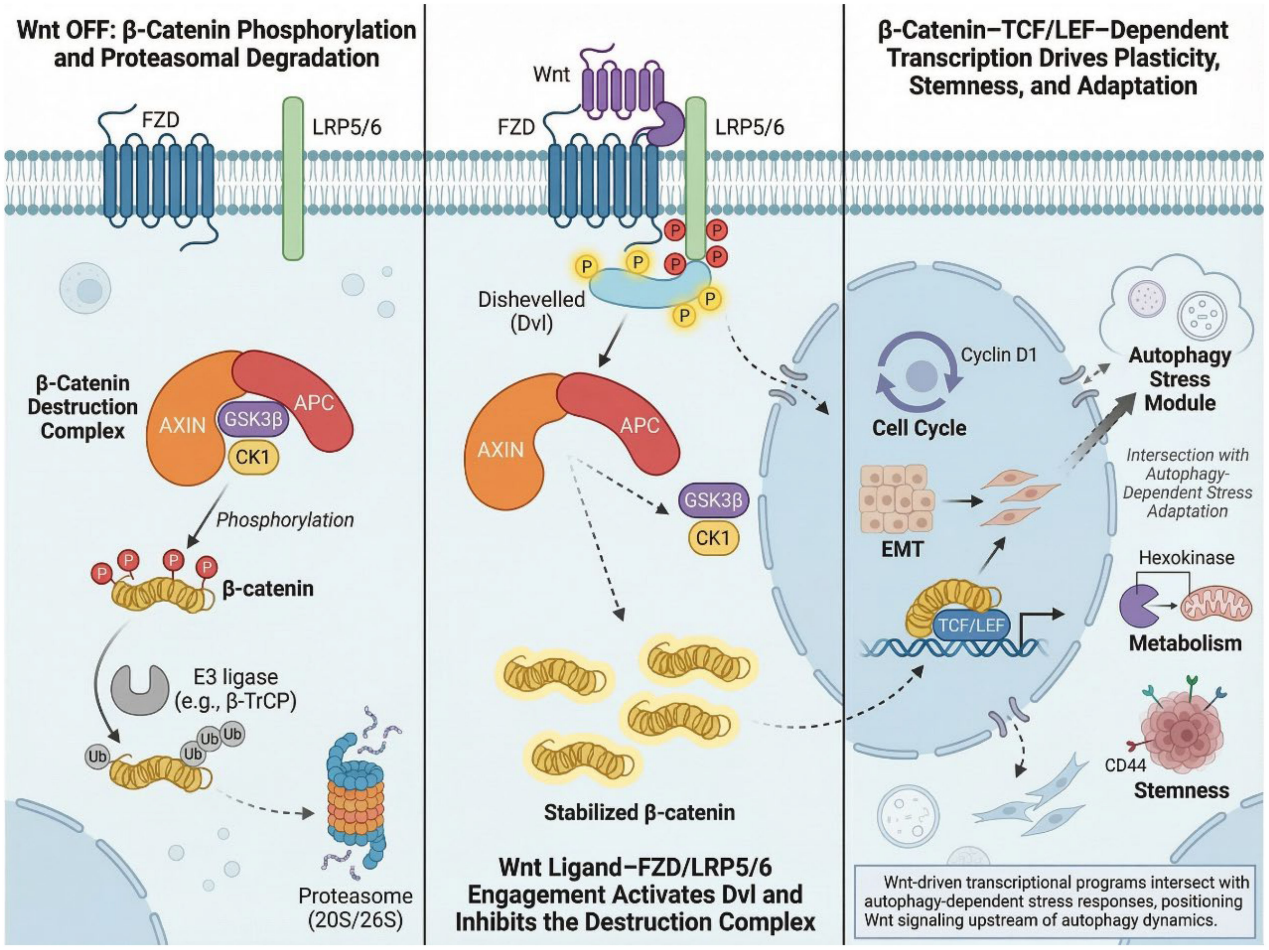
Canonical Wnt/β-catenin signaling and its intersection with autophagy-related stress adaptation in breast cancer. In the Wnt-OFF state, β-catenin is phosphorylated by the AXIN–APC–GSK3β–CK1 destruction complex, ubiquitinated by E3 ligases such as β-TrCP, and degraded by the proteasome. Upon Wnt ligand binding to FZD and LRP5/6, DVL is activated and the destruction complex is inhibited, leading to β-catenin stabilization and nuclear translocation. Nuclear β-catenin cooperates with TCF/LEF to induce transcriptional programs that regulate cell-cycle progression, EMT, metabolism, stemness, and stress adaptation. These Wnt-driven programs may intersect with autophagy-dependent responses, positioning Wnt signaling as an upstream regulator of autophagy dynamics in breast cancer.

The canonical Wnt/β-catenin signaling pathway is a central regulator of breast cancer development and progression, exerting profound effects on cell proliferation, lineage plasticity, metabolic adaptation, and stemness (31–36). In the absence of Wnt ligands, cytoplasmic β-catenin is continuously phosphorylated by the destruction complex composed of AXIN, APC, GSK3β, and CK1, targeting it for ubiquitin-mediated proteasomal degradation. Binding of Wnt ligands to Frizzled (FZD) receptors and the co-receptors LRP5/6 disrupts the destruction complex through Dishevelled (Dvl) activation, leading to β-catenin stabilization and nuclear accumulation. Nuclear β-catenin associates with TCF/LEF transcription factors to activate a transcriptional program that includes genes governing cell-cycle progression, epithelial–mesenchymal transition (EMT), metabolic reprogramming, and cancer stem cell (CSC) maintenance. In breast cancer, aberrant activation of canonical Wnt/β-catenin signaling is particularly prominent in triple-negative subtypes and stem-like tumor cell populations (37–42). This pathway promotes self-renewal capacity, enhances cellular plasticity, and supports adaptation to hostile microenvironmental conditions, thereby predisposing tumors to therapeutic resistance and disease recurrence. Importantly, many of these Wnt-driven phenotypes intersect functionally with autophagy-dependent stress responses, positioning Wnt signaling as a potential upstream determinant of autophagy dynamics rather than a parallel pathway. **Figure 1** shows the canonical Wnt/β-catenin signaling pathway and its role in regulating breast cancer cell plasticity and stress adaptation.

### 2.2 The mTOR axis and initiation of autophagy

mTOR functions as a master integrator of growth factor signaling, nutrient availability, and cellular energy status, and represents the principal gatekeeper of autophagy initiation. Acting as a central metabolic rheostat, mTOR translates extracellular cues and intracellular metabolic states into coordinated anabolic or catabolic responses. mTOR complex 1 (mTORC1), which is frequently activated downstream of PI3K–AKT signaling and other oncogenic pathways in breast cancer, directly suppresses autophagy by phosphorylating components of the ULK1 complex, thereby preventing autophagosome formation under nutrient-replete and growth-promoting conditions (43). This inhibitory control ensures that autophagy is restrained when biosynthetic programs dominate cellular behavior. In contrast, inhibition of mTORC1 activity—induced by nutrient deprivation, energetic stress, hypoxia, or pharmacological intervention—releases ULK1 from repression and triggers autophagy initiation. Importantly, this transition does not merely represent a binary on–off switch. Instead, the degree and duration of mTORC1 inhibition critically influence the magnitude and functional orientation of autophagy, determining whether autophagy serves as a transient adaptive response or progresses toward sustained flux with potential cytotoxic consequences. Such graded control is particularly relevant in cancer cells, which often exist in intermediate metabolic states rather than classical starvation conditions. Beyond early autophagosome formation, mTOR exerts multilayered control over later stages of the autophagy process by regulating lysosomal biogenesis, positioning, and degradative capacity. mTORC1 negatively regulates the nuclear translocation of transcription factor EB (TFEB), a master regulator of lysosomal and autophagy-related gene expression (44–47). When mTORC1 activity is suppressed, TFEB translocates to the nucleus and activates a coordinated transcriptional program that expands lysosomal mass, enhances lysosomal acidification, and sustains autophagic flux. This regulatory axis highlights that effective autophagy depends not only on autophagosome generation but also on lysosomal competence, a factor frequently altered in cancer cells exposed to chronic stress or therapeutic pressure.

Collectively, these mechanisms underscore that mTOR signaling governs both the initiation and the completion of autophagy, thereby shaping the efficiency, duration, and biological outcome of the autophagic process. This distinction has critical implications for experimental interpretation and therapeutic targeting. In breast cancer models, increased autophagosome abundance—often inferred from elevated LC3-II levels—may reflect either enhanced autophagy induction or impaired autophagosome degradation due to lysosomal dysfunction (48, 49). Similarly, accumulation of p62/SQSTM1 may indicate defective autophagic flux rather than suppression of autophagy initiation. These scenarios carry fundamentally different biological meanings and therapeutic implications. Therefore, accurate evaluation of autophagy in breast cancer requires assessment of autophagic flux, rather than reliance on static markers alone. Integrating flux assays with analyses of mTOR activity, TFEB localization, and lysosomal function is essential to determine whether autophagy operates as a cytoprotective stress-buffering mechanism or as a pathway capable of limiting tumor growth and plasticity. This mTOR-centered framework provides a mechanistic foundation for understanding why autophagy exhibits context-dependent roles in breast cancer and why its therapeutic modulation must be carefully aligned with upstream signaling states.

### 2.3 Wnt–mTOR–autophagy coupling models in breast cancer

Based on available evidence from breast cancer and related cancer models, we propose three working models of Wnt–mTOR–autophagy coupling. These models should be viewed as conceptual frameworks rather than uniformly validated mechanisms across all breast cancer subtypes. Emerging evidence supports the concept that Wnt/β-catenin and mTOR signaling converge to form a regulatory axis that dictates the directionality and functional outcome of autophagy in breast cancer. Rather than acting as parallel pathways, Wnt and mTOR function as integrated signaling hubs that determine whether autophagy is suppressed, activated, or repurposed toward distinct cellular outcomes. Based on current findings, three non-mutually exclusive coupling models can be proposed to conceptualize this dynamic crosstalk. In the first model, sustained activation of Wnt/β-catenin signaling reinforces mTORC1 activity, leading to suppression of autophagy initiation and limitation of lysosomal capacity. This configuration favors a highly anabolic and stress-tolerant cellular state characterized by enhanced survival, maintenance of cancer stem cell (CSC) traits, and resistance to therapy, and is frequently observed in stem-like and drug-resistant tumor populations. In contrast, pharmacological or genetic inhibition of Wnt signaling can attenuate mTORC1 activity, thereby relieving autophagy repression and restoring ULK1-dependent autophagic flux (50–53). In this second model, autophagy induction contributes to growth inhibition, apoptosis, or autophagic cell death, ultimately impairing tumor progression and CSC maintenance. However, the functional outcome of autophagy in this context remains highly context-dependent and may shift toward cytoprotection under alternative metabolic or genetic conditions. A third model introduces autophagy as an active regulator of Wnt signaling itself, whereby selective autophagic degradation of pathway components such as Dishevelled establishes a negative feedback loop that constrains excessive Wnt/β-catenin activation. Collectively, these models provide a unified framework in which Wnt/β-catenin signaling actively determines autophagy orientation through mTOR-dependent and -independent mechanisms, with important implications for therapeutic strategies targeting tumor cells and immune components within the tumor immune microenvironment. Importantly, the strength of evidence differs among these models. The ability of Wnt signaling to activate mTORC1 and thereby suppress autophagy is supported by mechanistic studies in several cancer and non-cancer systems. In breast cancer, evidence is stronger for Wnt-associated stemness, therapy resistance, and altered autophagy markers, whereas direct demonstration that sustained Wnt activation induces functional autophagic flux remains limited. Therefore, we distinguish Wnt-driven autophagy repression from stress-induced protective autophagy occurring in Wnt-active tumor cells.

### 2.4 Wnt–autophagy crosstalk beyond breast cancer

Wnt–autophagy crosstalk is not restricted to breast cancer. In several tumor and non-tumor contexts, Wnt/β-catenin signaling and autophagy form reciprocal regulatory loops that influence cell survival, differentiation, tissue remodeling, and stress adaptation (54). In colorectal cancer, aberrant Wnt/β-catenin activation is a dominant oncogenic event, and autophagy has been reported to either support tumor cell survival under metabolic stress or constrain Wnt signaling through selective degradation of pathway components (55–57). In hepatocellular, pancreatic, prostate, lung, and glioblastoma models, Wnt signaling frequently intersects with PI3K–AKT–mTOR activity, thereby influencing autophagy initiation, lysosomal function, and therapy resistance (14, 23, 58). Conversely, autophagy-related proteins can regulate Wnt output by controlling the turnover of Dishevelled, β-catenin-related complexes, or other signaling intermediates (57, 59). Beyond cancer, Wnt–autophagy interactions have also been implicated in tissue homeostasis, neurodegeneration, metabolic stress, fibrosis, and inflammatory diseases (54, 60, 61). These broader observations support the general principle that Wnt signaling and autophagy do not function as isolated pathways but instead cooperate or antagonize each other in a context-dependent manner. However, the biological consequences of this crosstalk are disease- and cell-type-specific. In breast cancer, its significance is particularly evident in Wnt-high stem-like tumor states, mTOR-dependent autophagy regulation, therapy-induced stress adaptation, and myeloid-mediated immune suppression. Thus, insights from other disease models provide a conceptual foundation, whereas breast cancer-specific validation remains essential for defining therapeutic relevance.

## 3 Reverse regulation: autophagy as a brake on Wnt signaling

While Wnt/β-catenin signaling is increasingly recognized as an upstream determinant of autophagy dynamics, accumulating evidence indicates that autophagy can, in turn, directly modulate Wnt pathway activity. This reciprocal regulation challenges the traditional view of autophagy as a passive downstream stress response and instead positions it as an active signaling modulator that fine-tunes oncogenic pathway output. Such bidirectional crosstalk is particularly relevant in breast cancer, where precise control of Wnt signaling amplitude and duration is critical for balancing proliferation, differentiation, and stress adaptation.

### 3.1 Autophagy-mediated turnover of Wnt signaling components

**Figure 2:**
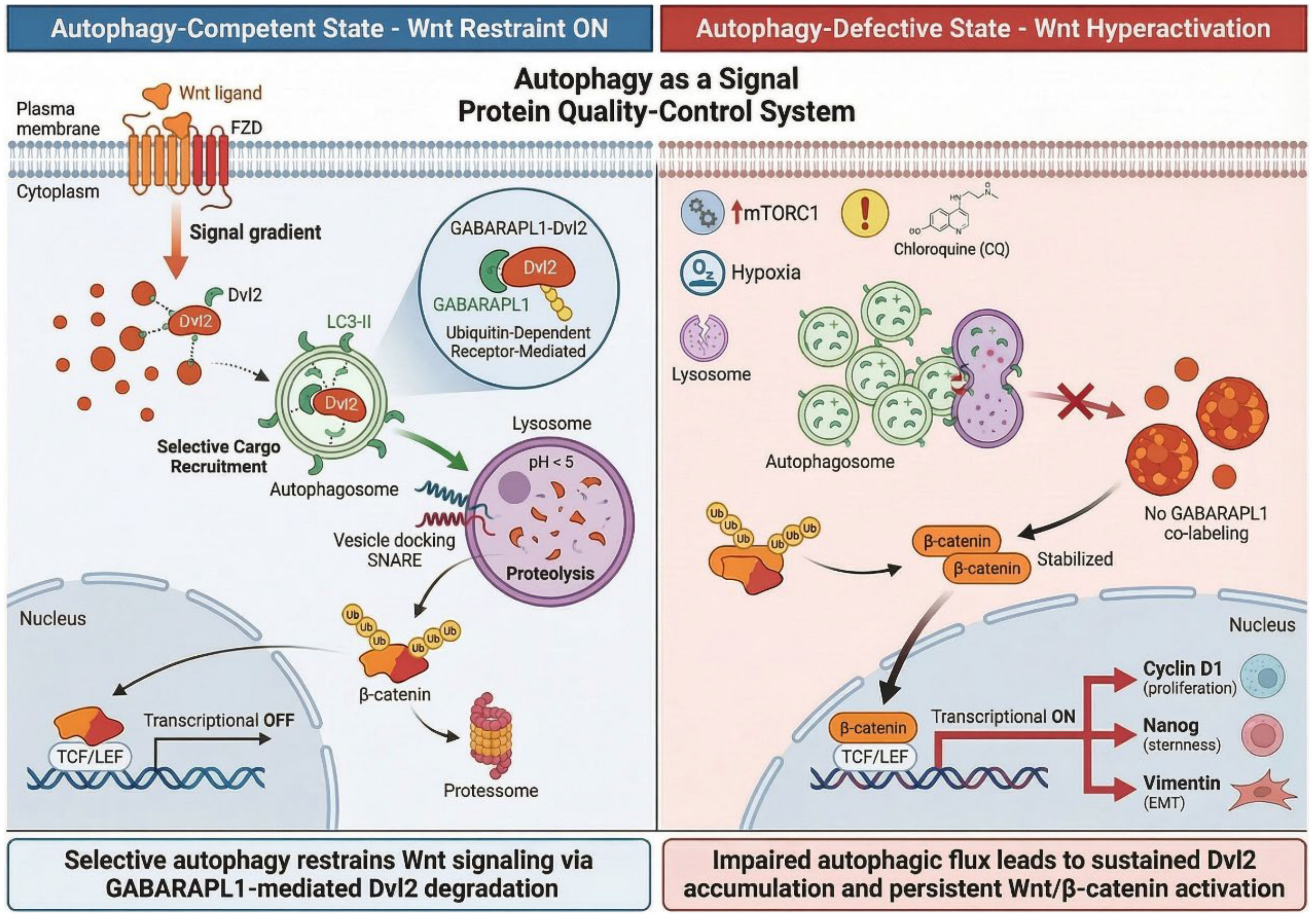
Selective autophagy restrains Wnt/β-catenin signaling through GABARAPL1-mediated Dvl2 degradation. In the autophagy-competent state, Dvl2 is selectively recruited into autophagosomes through GABARAPL1-associated cargo recognition and subsequently delivered to lysosomes for degradation. This process limits Dvl2 accumulation, promotes β-catenin proteasomal degradation, and restrains TCF/LEF-dependent transcription. In contrast, autophagy-defective conditions caused by mTORC1 hyperactivation, hypoxia, lysosomal dysfunction, or chloroquine-mediated flux blockade impair Dvl2 clearance. Accumulated Dvl2 sustains β-catenin stabilization and activates transcriptional programs associated with proliferation, stemness, and EMT.

One of the most compelling mechanisms by which autophagy constrains Wnt/β-catenin signaling involves the selective degradation of key pathway components rather than indiscriminate bulk cytoplasmic turnover. GABARAPL1, a member of the ATG8 family, has been shown to negatively regulate Wnt signaling by mediating the autophagic degradation of Dishevelled 2 (Dvl2), a central scaffold protein required for signal propagation downstream of Frizzled receptors (59)(**Figure 2**). Through facilitating the sequestration of Dvl2 into autophagosomes and its subsequent lysosomal degradation, GABARAPL1 effectively limits β-catenin stabilization and transcriptional activity. This mechanism represents an important conceptual advance by establishing autophagy as a signal protein quality-control system, capable of selectively targeting oncogenic signaling intermediates rather than functioning solely as a non-specific recycling pathway. In breast cancer cells, loss or downregulation of GABARAPL1 compromises this regulatory checkpoint, resulting in sustained accumulation of Dvl2 and persistent activation of Wnt/β-catenin signaling. Such deregulation promotes proliferative capacity, cellular plasticity, and tumorigenic potential, whereas restoration of autophagy competence enhances Dvl2 clearance and dampens Wnt-driven oncogenic programs. Importantly, autophagy-mediated turnover of Dvl2 introduces a layer of temporal and quantitative control over Wnt signaling that is fundamentally distinct from genetic ablation or pharmacological pathway inhibition (62–64). Rather than enforcing an abrupt on–off switch, selective autophagy enables graded modulation of signaling amplitude and duration, allowing dynamic adjustment of Wnt output in response to cellular stress, metabolic state, and microenvironmental fluctuations. This fine-tuning is particularly relevant in breast cancer, where tumor cells must continuously balance proliferative signaling with stress-adaptive programs. The effectiveness of this regulatory brake is critically dependent on intact autophagic flux and lysosomal function. Conditions that impair late-stage autophagy—such as chronic mTOR hyperactivation, hypoxia-associated lysosomal stress, or therapeutic lysosomal inhibition—may uncouple autophagosome formation from cargo degradation. Under these circumstances, apparent accumulation of autophagy markers may coexist with defective clearance of Dvl2, paradoxically reinforcing sustained Wnt/β-catenin activity. This provides a mechanistic explanation for why elevated LC3 or p62 levels do not necessarily indicate functional suppression of oncogenic signaling.

Collectively, these findings underscore how autophagy-mediated proteostasis directly intersects with signal transduction to restrain excessive Wnt pathway activation. At the same time, the inherent flexibility of this regulatory mechanism—while advantageous for cancer cells navigating fluctuating microenvironments—creates context-dependent vulnerabilities. Exploiting these vulnerabilities will require therapeutic strategies that align modulation of autophagy with the functional state of Wnt signaling and autophagic flux, rather than indiscriminate activation or inhibition of the autophagy machinery.

### 3.2 Beclin-1/BECN1 and WNT1-driven tumorigenesis: context-dependent suppression

**Figure 3:**
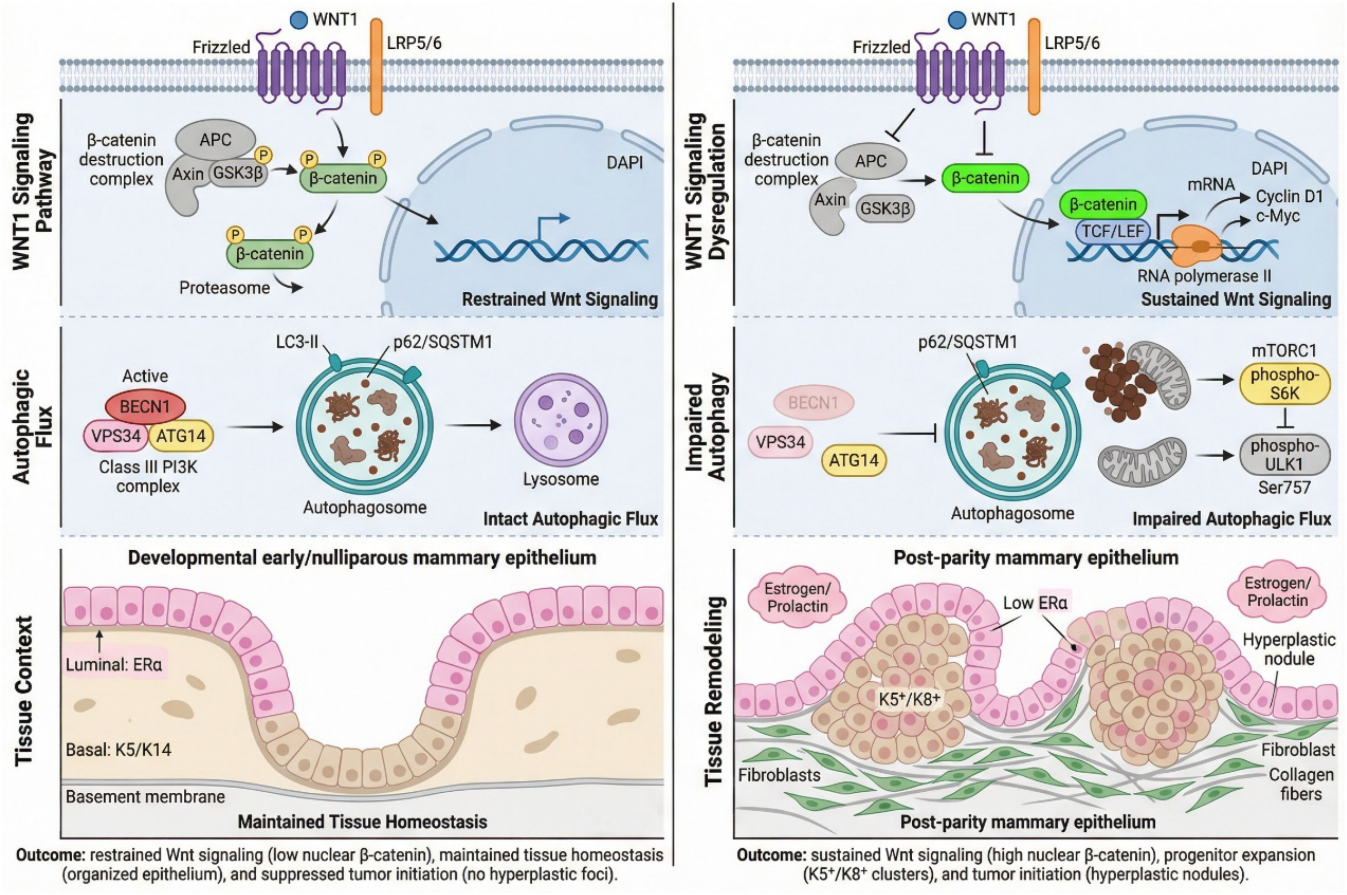
BECN1-dependent autophagy constrains WNT1-driven mammary tumorigenesis in a tissue-context-dependent manner. In developmental or nulliparous mammary epithelium, intact BECN1–VPS34–ATG14-dependent autophagic flux supports β-catenin turnover, restrains WNT1 signaling, and helps maintain organized epithelial homeostasis. In contrast, impaired BECN1-dependent autophagy disrupts autophagic flux, enhances mTORC1 activity, inhibits ULK1, and permits sustained β-catenin–TCF/LEF transcription. In the post-parity mammary epithelium, this dysregulated state is associated with progenitor expansion, stromal remodeling, hyperplastic nodules, and increased tumor initiation.

Beyond the selective degradation of individual signaling intermediates, core autophagy regulators can modulate Wnt-driven tumorigenesis at a broader, systems level. Beclin-1 (BECN1), a central initiator of autophagosome formation and a haploinsufficient tumor suppressor in breast cancer, has been shown to exert inhibitory effects on mammary tumorigenesis driven by WNT1 activation. Evidence from genetically engineered mouse models demonstrates that intact BECN1-dependent autophagy constrains WNT1-induced tumor initiation and progression, indicating that functional autophagy machinery can antagonize oncogenic Wnt signaling during early stages of tumor development (65). However, the interaction between BECN1, autophagy, and Wnt signaling is highly context-dependent rather than universally tumor suppressive. While BECN1-mediated autophagy limits tumorigenesis in WNT1-driven models, its impact varies with developmental stage, hormonal status, and cellular differentiation state. Notably, parity-associated remodeling of the mammary gland alters the biological consequences of WNT1 activation and modulates the tumor-suppressive effect of BECN1, suggesting that autophagy intersects with Wnt signaling within a temporally and physiologically regulated framework (66–70). These findings underscore that autophagy does not simply counteract oncogenic signaling in a static manner, but instead integrates developmental cues and tissue context to shape disease trajectories. At a mechanistic level, BECN1-dependent autophagy likely constrains Wnt-driven tumorigenesis by maintaining cellular homeostasis and preventing the stabilization of aberrant signaling states during periods of heightened proliferative or developmental plasticity (71–76). Loss or attenuation of BECN1 compromises autophagic flux and lysosomal capacity, potentially allowing sustained Wnt pathway output, accumulation of oncogenic intermediates, and reinforcement of stem-like or progenitor cell states. In this setting, Wnt signaling may escape normal homeostatic constraints, facilitating malignant transformation and progression. Importantly, these observations highlight a critical principle: the tumor-suppressive versus tumor-supportive roles of autophagy cannot be inferred from the activity of core autophagy regulators alone, but must be interpreted within the broader signaling, developmental, and microenvironmental context. In breast cancer, such context dependency provides a unifying explanation for the divergent roles of autophagy observed across molecular subtypes and disease stages, as well as for the heterogeneity in therapeutic responses to autophagy modulation (77–80). Understanding how BECN1-dependent autophagy interfaces with Wnt signaling across physiological and pathological states will therefore be essential for designing rational strategies that exploit autophagy as a therapeutic vulnerability rather than a blunt intervention. **Figure 3** shows the context-dependent role of BECN1-dependent autophagy in regulating WNT1-driven mammary tumorigenesis.

## 4 Cell-state outcomes: how the Wnt–mTOR–autophagy axis shapes tumor cell states

The functional consequences of Wnt–mTOR–autophagy crosstalk ultimately manifest at the level of tumor cell states, including stemness, plasticity, invasive capacity, and intratumoral heterogeneity. Rather than exerting uniform effects, this regulatory axis dynamically sculpts breast cancer cell phenotypes in a context-dependent manner, thereby influencing disease progression and therapeutic responsiveness.

### 4.1 Stemness and cancer stem cell traits

**Figure 4:**
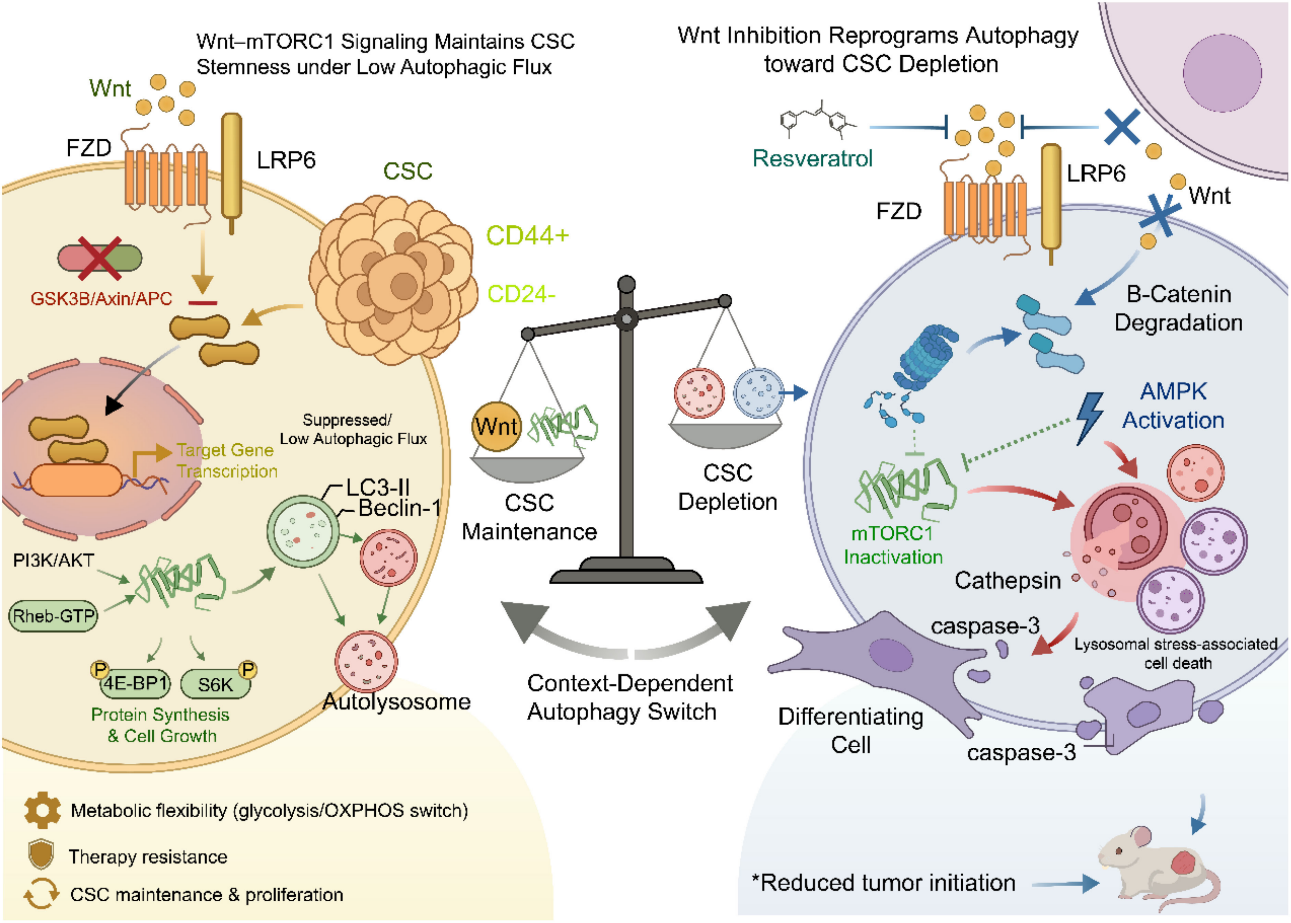
Context-dependent Wnt–mTOR–autophagy regulation of breast cancer stem-like cells. Sustained Wnt/β-catenin signaling activates mTORC1 and is associated with low autophagic flux, metabolic flexibility, CSC maintenance, and therapy resistance. In contrast, Wnt inhibition may relieve mTORC1-mediated repression and reprogram autophagy toward lysosomal stress, apoptosis, CSC depletion, and reduced tumor initiation.

Canonical Wnt/β-catenin signaling is a well-established driver of breast cancer stem cell (BCSC) maintenance, self-renewal, and tumor-initiating capacity. Elevated Wnt activity sustains transcriptional programs that reinforce pluripotency-associated genes, metabolic flexibility, and resistance to stress, all of which are hallmarks of CSC populations. Autophagy intersects with this process in a bidirectional manner, functioning either as a supportive mechanism that preserves stemness under adverse conditions or as a restrictive force that limits CSC maintenance when dysregulated or excessively activated. In stem-like breast cancer cells, basal autophagy often serves a cytoprotective role by buffering metabolic stress and maintaining mitochondrial homeostasis, thereby facilitating long-term self-renewal. However, suppression of Wnt signaling can shift this balance by altering mTOR activity and autophagic flux. For example, resveratrol-mediated inhibition of Wnt/β-catenin signaling induces autophagy and concomitantly reduces BCSC frequency and stemness-associated traits (81)(**Figure 4**). In this context, autophagy induction is coupled to loss of β-catenin-driven transcriptional programs, leading to impaired sphere formation and diminished tumor-initiating potential. These findings illustrate a key principle: the impact of autophagy on CSC traits is not intrinsic to autophagy itself but is dictated by the upstream Wnt–mTOR signaling context. In tumors with sustained Wnt/β-catenin activation, autophagy is not uniformly activated. Instead, Wnt-driven mTORC1 activity may suppress canonical autophagy initiation and lysosomal function, thereby supporting an anabolic, stem-like and therapy-resistant state. However, under therapeutic or metabolic stress, residual or compensatory autophagy may be engaged as a cytoprotective mechanism in Wnt-active tumor cells. Thus, the relationship between Wnt activity and autophagy depends on whether autophagy is mTOR-repressed, stress-induced, or functionally required for survival (**Figure 4**).

### 4.2 EMT, invasion, and metastatic programs

**Figure 5:**
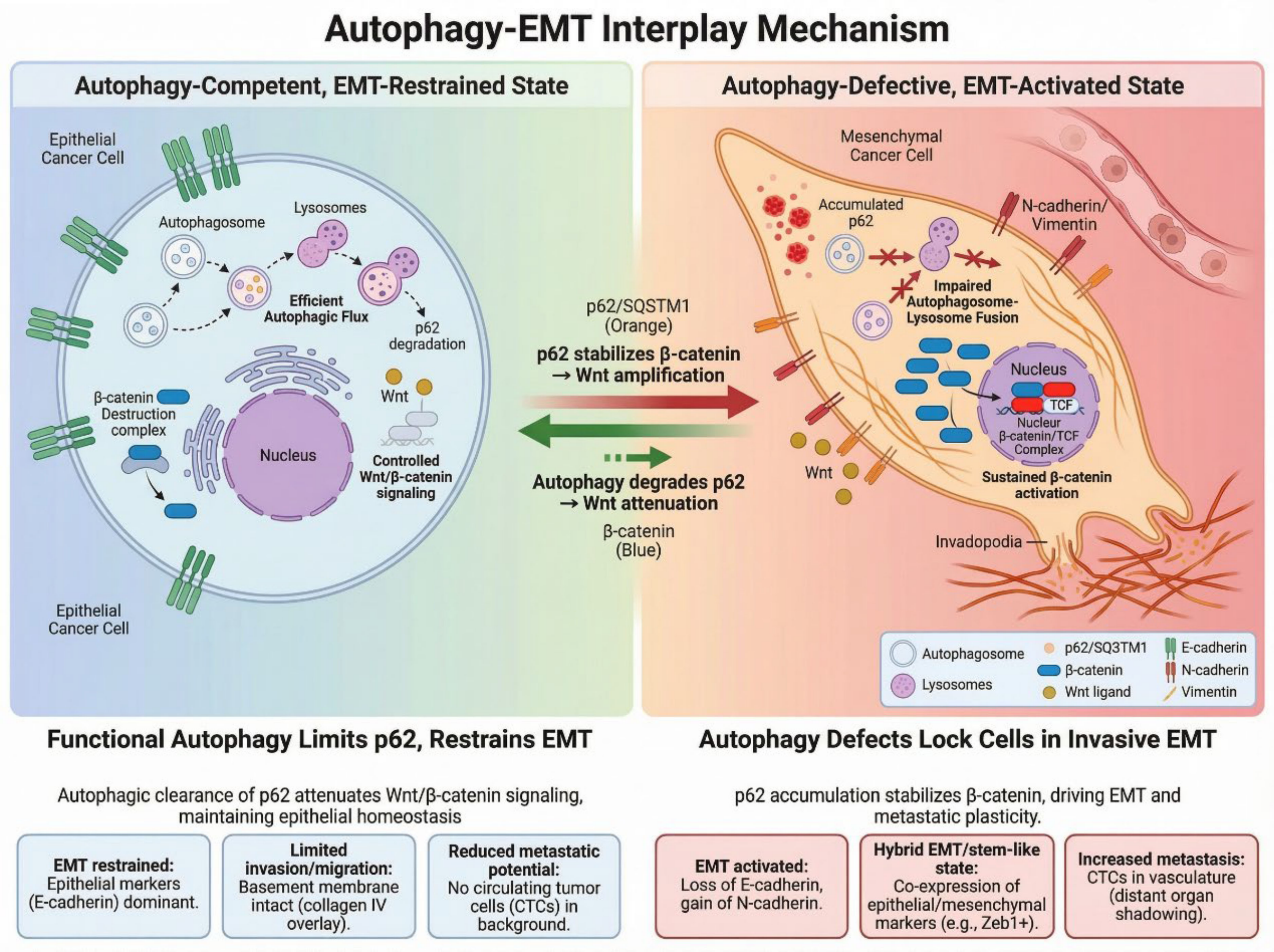
Autophagy–EMT interplay through p62/SQSTM1-mediated regulation of Wnt/β-catenin signaling. In the autophagy-competent state, efficient autophagic flux promotes p62/SQSTM1 degradation, restrains β-catenin stabilization, and limits Wnt/β-catenin signaling. This maintains epithelial identity, characterized by E-cadherin dominance, preserved basement membrane integrity, limited invasion, and reduced metastatic potential. In contrast, impaired autophagosome–lysosome fusion leads to p62/SQSTM1 accumulation, which may stabilize β-catenin and amplify Wnt signaling. In Wnt-responsive breast cancer contexts, sustained β-catenin–TCF activity can promote EMT, loss of epithelial features, gain of N-cadherin and vimentin, invadopodia formation, and hybrid EMT/stem-like states. However, metastatic dissemination should not be viewed as uniformly Wnt-high, because recent evidence in colorectal cancer shows that MAPK-high–WNT-low transcriptional states can also drive metastasis. Thus, Wnt–autophagy coupling represents a context-dependent plasticity axis rather than a universal requirement for metastatic spread.

Beyond stemness, the Wnt–autophagy axis plays a pivotal role in regulating epithelial–mesenchymal transition (EMT), invasion, and metastatic dissemination. Wnt/β-catenin signaling is a central inducer of EMT, driving transcriptional programs that repress epithelial identity while promoting mesenchymal features, migratory capacity, and invasive behavior. Autophagy interfaces with EMT signaling at multiple levels, including regulation of cytoskeletal dynamics, turnover of adhesion molecules, and control of signaling intermediates. A key molecular node linking autophagy and Wnt-driven EMT is the autophagy adaptor p62/SQSTM1 (82). Accumulation of p62, often indicative of impaired autophagic flux, has been associated with sustained β-catenin activation and reinforcement of EMT and stem-like programs. Conversely, efficient autophagic degradation of p62 can attenuate Wnt signaling output, thereby restraining EMT progression (**Figure 5**). This establishes a mechanistic logic in which autophagy competency modulates the amplitude of Wnt-driven EMT through regulation of p62 and β-catenin stability. Through this coupling, defects in autophagic flux may lock tumor cells into a highly invasive, plastic state characterized by concurrent EMT and stemness features. Such hybrid states are increasingly recognized as particularly aggressive and therapy-resistant, underscoring the importance of autophagy–Wnt interplay in metastatic progression.

However, the relationship between Wnt/β-catenin activity and metastatic dissemination should not be interpreted as universally linear or unidirectional. A recent Nature Cancer study in colorectal cancer demonstrated that a highly metastatic MAPK-high–WNT-low transcriptional state can drive metastatic dissemination, in which chromosomal amplifications of MAPK pathway genes increased MAPK activity while suppressing WNT-associated transcriptional programs, including stem cell genes (83). Pharmacological inhibition of mutant KRAS^G12D reduced this MAPK-high–WNT-low state and decreased both lung and liver metastases. Although this evidence was obtained in colorectal cancer rather than breast cancer, it provides an important conceptual caution: Wnt signaling may promote EMT, stemness, and invasion in some tumor contexts, but high Wnt activity is not invariably required for metastatic dissemination. Instead, metastatic competence may arise from dynamic pathway rewiring, including MAPK-dominant and WNT-suppressed cell states. Therefore, in breast cancer, Wnt–autophagy coupling should be interpreted as a context-dependent regulator of plasticity and invasion rather than as a universal driver of all metastatic programs.

### 4.3 Intratumoral heterogeneity: a single-cell perspective

The influence of the Wnt–mTOR–autophagy axis on tumor cell states is further amplified by intratumoral heterogeneity. Single-cell transcriptomic analyses have revealed that breast tumors comprise diverse cellular subpopulations with distinct levels of Wnt signaling activity, autophagy-related gene expression, and stress-response programs. These heterogeneous configurations cannot be fully captured by bulk analyses but critically shape therapeutic outcomes. At the single-cell level, subsets of tumor cells with high Wnt/β-catenin activity frequently exhibit altered expression of autophagy regulators, including p62/SQSTM1 and ATG family members, suggesting differential reliance on autophagy for survival and plasticity. Other subpopulations display enhanced autophagic signatures concomitant with reduced Wnt signaling, potentially reflecting differentiated or therapy-sensitive states. Such heterogeneity in Wnt–autophagy coupling provides a mechanistic explanation for variable treatment responses within the same tumor, as distinct cell states may differentially engage cytoprotective or cytotoxic autophagy programs. Importantly, this cell-state diversity implies that therapeutic modulation of autophagy or Wnt signaling will not exert uniform effects across all tumor cells. Instead, treatment may selectively eliminate certain subpopulations while sparing others, potentially driving adaptive resistance. Integrating single-cell and spatial profiling approaches will therefore be essential to map Wnt–mTOR–autophagy configurations across tumor ecosystems and to design combination strategies that effectively target heterogeneous cell states.

## 5 TIME layer: Wnt–mTOR–autophagy signaling in myeloid-driven immunosuppression

While most studies on Wnt–mTOR–autophagy crosstalk in breast cancer have focused on tumor cell–intrinsic processes, emerging evidence indicates that this regulatory axis also plays a pivotal role in shaping the tumor immune microenvironment (TIME). In particular, autophagy-dependent regulation of myeloid cell fate and function is increasingly recognized as a critical determinant of immune suppression, tumor progression, and therapeutic resistance (84–86).

### 5.1 Myeloid-derived suppressor cells and early-stage MDSCs in breast cancer

Myeloid-derived suppressor cells (MDSCs) represent a heterogeneous population of immature myeloid cells with potent immunosuppressive activity (87–91). In breast cancer, expansion and accumulation of MDSCs within tumors and peripheral compartments correlate with disease progression, metastatic potential, and poor clinical outcomes. MDSCs suppress antitumor immunity through multiple mechanisms, including inhibition of cytotoxic T cell proliferation and function, promotion of regulatory T cell expansion, depletion of essential amino acids, and production of immunosuppressive cytokines and reactive oxygen species. Recent studies have highlighted the importance of early-stage MDSCs (eMDSCs) as a precursor population that sustains the immunosuppressive myeloid pool (92–95). Unlike terminally differentiated suppressor subsets, eMDSCs retain high plasticity and survival capacity, allowing them to adapt dynamically to tumor-derived signals. Their persistence within the TIME provides a continuous source of immunosuppressive myeloid cells, thereby maintaining an immune-suppressive niche favorable for tumor growth. Understanding the signaling mechanisms that govern eMDSC survival and differentiation is therefore critical for deciphering immune escape in breast cancer.

### 5.2 SOCS3 deficiency–driven Wnt/mTOR activation represses autophagy and promotes eMDSC survival

**Figure 6:**
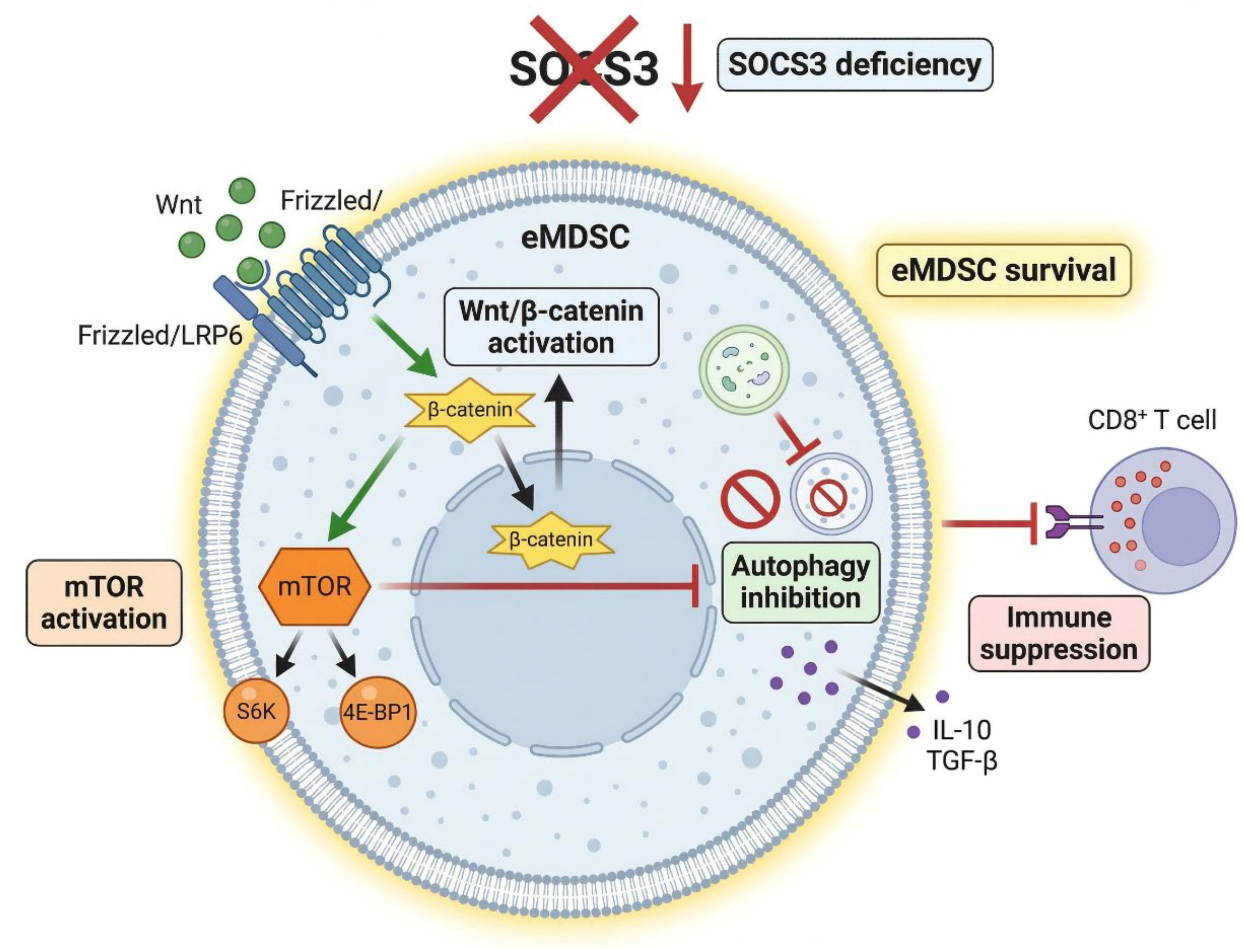
SOCS3 deficiency activates the Wnt/β-catenin–mTOR axis and suppresses autophagy in early-stage MDSCs. SOCS3 deficiency in early-stage myeloid-derived suppressor cells (eMDSCs) promotes Wnt/β-catenin signaling through Frizzled/LRP6-associated activation, leading to β-catenin accumulation and mTOR activation. Activated mTOR enhances downstream S6K and 4E-BP1 signaling while inhibiting autophagy, thereby supporting eMDSC survival. Autophagy-repressed eMDSCs produce immunosuppressive mediators such as IL-10 and TGF-β and suppress CD8^+^ T-cell activity, contributing to immune evasion in the breast cancer microenvironment.

A seminal mechanistic insight into the regulation of eMDSC fate has been provided by studies demonstrating that SOCS3 deficiency acts as a molecular switch linking Wnt/mTOR signaling to autophagy repression in myeloid cells (96). SOCS3, a well-known negative regulator of cytokine signaling, plays a crucial role in maintaining immune homeostasis. Loss or downregulation of SOCS3 in myeloid compartments disrupts this balance, leading to aberrant activation of downstream oncogenic and metabolic pathways. In SOCS3-deficient eMDSCs, activation of Wnt/β-catenin signaling and its downstream mTOR pathway results in sustained suppression of autophagy. This repression of autophagic flux enhances cellular survival, prevents differentiation or apoptotic clearance, and supports the long-term persistence of eMDSCs within the TIME. Functionally, autophagy-deficient eMDSCs exhibit heightened immunosuppressive capacity, effectively dampening antitumor T cell responses and promoting tumor progression (26, 28, 97). This mechanism establishes a direct causal link between SOCS3 loss, Wnt/mTOR hyperactivation, autophagy inhibition, and myeloid-mediated immune suppression. Importantly, it extends the functional relevance of the Wnt–mTOR–autophagy axis beyond tumor cells, positioning it as a central regulator of immune cell fate within the breast cancer microenvironment. **Figure 6** schematically illustrates how SOCS3 deficiency reprograms early-stage myeloid-derived suppressor cells (eMDSCs) through activation of the Wnt/β-catenin–mTOR signaling axis. These findings also provide a mechanistic explanation for how tumors exploit conserved developmental and metabolic pathways to stabilize an immunosuppressive niche. Mechanistically, the link between autophagy inhibition and increased immunosuppressive mediator production in eMDSCs is likely to involve several inflammation- and metabolism-associated transcriptional programs. SOCS3 deficiency can enhance cytokine-dependent JAK–STAT signaling, particularly STAT3 activity, which is a central regulator of MDSC expansion, survival, and suppressive function. In parallel, mTOR activation may promote HIF-1α- and C/EBPβ-associated myeloid programs, while impaired autophagic flux may lead to p62/SQSTM1 accumulation and sustained NF-κB-related inflammatory signaling. These pathways may collectively support the production of IL-10 and TGF-β and reinforce the immunosuppressive phenotype of eMDSCs. However, the precise transcriptional cascade connecting autophagy repression to IL-10/TGF-β induction in breast cancer eMDSCs remains incompletely defined and requires further experimental validation. Importantly, activation of Wnt/mTOR signaling in MDSCs should not be interpreted as evidence that MDSCs are the dominant source of Wnt ligands within the breast cancer microenvironment. As this review did not perform a *de novo* quantitative analysis of breast cancer single-cell RNA-seq datasets, we do not report fold differences in Wnt ligand expression between tumor cells and MDSCs. Instead, available evidence is interpreted to indicate that Wnt/mTOR signaling may operate within myeloid cells in a context-dependent manner, whereas the relative contribution of tumor-derived, stromal-derived, and myeloid-derived Wnt ligands remains to be defined by future cell-type-resolved and spatial analyses.

### 5.3 Autophagy as an immunometabolic fate switch in the tumor immune microenvironment

The SOCS3–Wnt–mTOR–autophagy axis in eMDSCs highlights a broader conceptual framework in which autophagy functions as an immunometabolic fate switch. Rather than serving merely as a housekeeping process, autophagy integrates oncogenic signaling, metabolic stress, and cytokine cues to determine whether myeloid cells undergo differentiation, apoptosis, or acquire sustained immunosuppressive activity. Within the TIME, differential autophagy states across myeloid populations may dictate the balance between immune activation and immune suppression. Autophagy repression favors the survival and accumulation of immunosuppressive myeloid cells, whereas restoration of autophagic flux may destabilize these populations and enhance antitumor immunity (98–101). This perspective suggests that therapeutic modulation of autophagy—either directly or through upstream regulators such as Wnt and mTOR—could reprogram the immune microenvironment in favor of immune surveillance. Looking forward, a major challenge lies in resolving the spatial and cellular heterogeneity of Wnt–mTOR–autophagy signaling within the TIME. Integrating single-cell and spatial transcriptomic approaches with functional autophagy assays will be essential to map autophagy-dependent immune states and to identify patient subsets most likely to benefit from autophagy-targeted interventions. Such efforts will be critical for translating mechanistic insights into rational combination strategies that simultaneously target tumor cells and immune suppressive niches.

### 5.4 Tumor-associated macrophages as an additional Wnt-responsive and Wnt-producing myeloid compartment

In addition to MDSCs, tumor-associated macrophages (TAMs) represent a major myeloid population in breast cancer and have been strongly implicated in tumor invasion, angiogenesis, immune suppression, metastatic dissemination, and poor clinical outcome (102). Therefore, a myeloid-centered discussion of Wnt–mTOR–autophagy crosstalk should not be restricted to MDSCs. Earlier mechanistic work demonstrated that macrophage-associated Wnt5a signaling can promote breast cancer cell invasiveness by regulating tumor cell migration and proteolytic activity, supporting the concept that macrophage-derived or macrophage-associated Wnt signals may contribute to tumor progression (103, 104). However, the relative quantitative contribution of macrophage-derived Wnt ligands versus tumor-derived Wnt ligands remains incompletely defined, particularly at single-cell and spatial levels. Thus, TAM-derived Wnt should be discussed as a biologically plausible and experimentally supported component of the breast cancer microenvironment, but not as a quantitatively dominant source unless directly demonstrated by cell-type-resolved analyses.

### 5.4 Metabolic consequences of Wnt–mTOR–autophagy crosstalk in the TIME

If autophagy functions as an immunometabolic fate switch in the breast cancer TIME, its functional state should ideally be linked to metabolic readouts such as extracellular acidification rate (ECAR), oxygen consumption rate (OCR), mitochondrial fitness, glycolytic dependency, and metabolite production. Direct studies connecting Wnt–mTOR–autophagy bidirectional crosstalk to ECAR/OCR changes in breast cancer MDSCs remain limited. However, related breast cancer studies support the broader principle that autophagy can regulate metabolic adaptation. For example, autophagy inhibition by chloroquine has been reported to target cancer stem cells in triple-negative breast cancer by inducing mitochondrial damage and impairing stress-adaptive survival programs (105). More recent work showed that leptin-induced autophagy supports mitochondrial ATP production in ER-positive and triple-negative breast cancer cells and contributes to glycolytic ATP production in triple-negative cells; chloroquine treatment reduced these metabolic outputs, indicating that autophagy can sustain both mitochondrial and glycolytic energy production in a context-dependent manner (106). These findings suggest that blocking cytoprotective autophagy may impair mitochondrial fitness and glycolytic adaptation in selected breast cancer states. In myeloid cells, metabolic regulation is equally important. MDSCs are metabolically heterogeneous and can rely on glycolysis, oxidative phosphorylation, fatty acid oxidation, amino-acid metabolism, and lipid accumulation depending on tumor context and differentiation state (107, 108). Single-cell studies in breast cancer have begun to define distinct MDSC-like myeloid populations, but direct single-cell measurements linking Wnt activity, mTOR signaling, autophagic flux, ECAR/OCR-equivalent metabolic states, and suppressive function remain insufficient. Therefore, we interpret the SOCS3–Wnt/mTOR–autophagy axis as a mechanistic framework rather than a fully metabolically validated model (109). Future studies should combine Seahorse-based ECAR/OCR assays, isotope tracing, metabolomics, single-cell RNA-seq, spatial transcriptomics, and functional autophagy-flux assays in sorted or spatially resolved MDSC subsets. Such studies would clarify whether autophagy blockade shifts MDSCs away from oxidative or glycolytic programs, whether autophagy induction destabilizes suppressive myeloid states, and how these changes influence CD8^+^ T-cell function.

## 6 Therapeutic translation: when to induce autophagy and when to block it

The dual and context-dependent roles of autophagy in breast cancer have long complicated efforts to therapeutically target this pathway. However, accumulating evidence from Wnt-targeted interventions suggests that these seemingly contradictory outcomes can be reconciled by considering the functional positioning of autophagy within the Wnt–mTOR signaling context. Rather than asking whether autophagy is universally beneficial or detrimental, a more clinically relevant question is when autophagy should be induced and when it should be inhibited to maximize therapeutic efficacy.

### 6.1 Strategy I: Wnt inhibition–induced autophagy as an anti-tumor mechanism

Multiple pharmacological and biological agents targeting Wnt/β-catenin signaling have been shown to induce autophagy and suppress breast cancer progression (36, 110–114). Thiouracil–triazole conjugates represent a class of small molecules that downregulate Wnt/β-catenin signaling and concomitantly activate autophagy, leading to growth inhibition and reduced viability in breast cancer cells (115). Similarly, kallistatin exerts antitumor effects by modulating Wnt signaling and microRNA synthesis, thereby triggering both autophagy and apoptosis (116). Natural compounds such as resveratrol further exemplify this paradigm, as Wnt inhibition–associated autophagy induction has been linked to depletion of breast cancer stem cell populations and impairment of tumor-initiating capacity (81). A critical consideration in this strategy is whether autophagy induction functions as a cytotoxic mechanism or merely accompanies other forms of cell death. In several Wnt-targeted settings, autophagy appears to cooperate with apoptotic pathways, amplifying antitumor effects rather than acting as a primary reminder of survival. However, reliance on static autophagy markers alone risks misinterpretation. Robust assessment of autophagic flux—using lysosomal inhibitors and functional assays—is essential to determine whether autophagy is truly activated and whether it contributes causally to tumor suppression. These studies collectively support the notion that Wnt inhibition–induced autophagy can be therapeutically advantageous when it drives or facilitates cell death and CSC depletion.

### 6.2 Strategy II: Blocking protective autophagy to sensitize therapy

In contrast to settings where autophagy induction is cytotoxic, numerous breast cancer models demonstrate that autophagy acts as a protective stress-adaptive mechanism, particularly under therapeutic pressure. In these contexts, autophagy enables tumor cells to withstand metabolic stress, DNA damage, and cytotoxic insults, thereby promoting drug resistance and disease persistence. A representative example is the combination of Wnt pathway inhibition with pharmacological blockade of autophagy. Antibody-coated nanoshells targeting Frizzled 7 (FZD7) suppress Wnt/β-catenin signaling in triple-negative breast cancer, but their therapeutic efficacy is markedly enhanced when combined with chloroquine, a lysosomal inhibitor that disrupts autophagic flux. This combinatorial approach illustrates a rational strategy in which Wnt inhibition destabilizes oncogenic signaling while concurrent autophagy blockade prevents the activation of compensatory survival programs. Genetic regulators further support this concept. BRMS1L has been shown to enhance chemosensitivity in breast cancer by inhibiting autophagy, with clinical correlations indicating improved response to neoadjuvant chemotherapy and favorable prognosis in patients with higher BRMS1L expression (117). Additional studies implicating ATG4A, N-Myc and STAT interactor (NMI), and traditional medicinal formulations reinforce the principle that suppression of protective autophagy can restore drug sensitivity (118). Collectively, these findings argue that autophagy inhibition is therapeutically beneficial when autophagy primarily serves a cytoprotective role downstream of oncogenic signaling.

### 6.3 A decision framework for context-specific autophagy modulation

Integrating these observations, a decision framework emerges in which therapeutic manipulation of autophagy is guided by Wnt signaling status, autophagy functionality, and cellular context. In tumors characterized by high Wnt/β-catenin activity, robust mTOR signaling, and evidence of autophagy-dependent survival or drug resistance, combined inhibition of Wnt signaling and autophagy may represent an optimal strategy. Conversely, in settings where Wnt inhibition unleashes autophagy that contributes to apoptosis or autophagic cell death, further promotion of lethal autophagy may enhance antitumor efficacy. Central to this framework is the need for biomarker-driven stratification. Indicators of Wnt pathway activation, autophagic flux competence, and stress-adaptive reliance on autophagy will be critical for identifying patients most likely to benefit from autophagy induction versus inhibition. Without such stratification, blanket modulation of autophagy risks counterproductive outcomes, including enhanced tumor survival or immune suppression.

## 7 Biomarkers and clinical stratification: toward actionable Wnt–autophagy–myeloid profiling

The context-dependent roles of Wnt–mTOR–autophagy crosstalk in breast cancer underscore the urgent need for biomarker-guided clinical stratification. Without reliable indicators of pathway activity and functional autophagy status, therapeutic modulation of autophagy risks producing unpredictable or even deleterious outcomes. Integrating biomarkers that capture oncogenic signaling, stress-adaptive capacity, and immune suppression may therefore be essential for translating mechanistic insights into precision treatment strategies.

### 7.1 Biomarkers of Wnt/β-catenin signaling activity

Assessment of Wnt/β-catenin pathway activation represents a foundational step in stratifying breast cancer patients for autophagy-targeted interventions (31, 34, 119–122). Nuclear localization of β-catenin, as detected by immunohistochemistry or immunofluorescence, remains a widely used and clinically accessible surrogate marker of canonical Wnt signaling activation. Beyond single-protein readouts, transcriptional signatures composed of Wnt target genes provide a more comprehensive measure of pathway output and may better capture intratumoral heterogeneity. In particular, gene expression panels reflecting β-catenin–TCF/LEF transcriptional activity can be derived from bulk RNA sequencing or single-cell transcriptomic datasets. These signatures may distinguish tumors driven by sustained Wnt activation from those with transient or context-dependent signaling, thereby informing whether Wnt inhibition is likely to disrupt stemness, metabolic adaptation, and downstream autophagy regulation. Importantly, Wnt pathway activity should be interpreted in conjunction with mTOR signaling status, as their convergence critically determines autophagy orientation.

### 7.2 Functional biomarkers of autophagy competence and flux

Given the dual nature of autophagy, accurate assessment of autophagic function is indispensable for therapeutic decision-making. Static markers such as LC3-II accumulation or p62/SQSTM1 levels provide initial insights into autophagy status but are insufficient to discriminate between enhanced autophagy initiation and impaired autophagic degradation. For instance, elevated p62 may reflect defective autophagic flux rather than suppressed autophagy induction, with distinct biological and therapeutic implications. Evaluation of transcription factor EB (TFEB) nuclear localization offers additional information regarding lysosomal biogenesis and autophagy capacity, linking upstream mTOR signaling to downstream degradative function. However, definitive characterization of autophagy requires dynamic assessment of autophagic flux, typically achieved through pharmacological blockade of lysosomal degradation using agents such as chloroquine or bafilomycin A1. Changes in LC3-II and p62 levels upon lysosomal inhibition provide functional evidence of flux competency and should be incorporated into both preclinical and translational studies. Together, these biomarkers enable classification of tumors into autophagy-competent versus autophagy-impaired states, and further into cytoprotective versus cytotoxic autophagy configurations when interpreted alongside Wnt–mTOR activity. Such stratification is essential for determining whether autophagy should be induced or inhibited in a given therapeutic context.

### 7.3 Immune microenvironment biomarkers: myeloid suppression signatures

Beyond tumor cell–intrinsic parameters, biomarkers reflecting the tumor immune microenvironment add a critical dimension to clinical stratification. Expansion of myeloid-derived suppressor cells (MDSCs), particularly early-stage MDSCs, is a hallmark of immune suppression in breast cancer and is closely linked to poor prognosis and therapeutic resistance (109, 123–127). Transcriptional signatures associated with MDSC abundance and suppressive function can be inferred from bulk or single-cell RNA sequencing and may serve as indicators of an immunosuppressive TIME. SOCS3 expression status emerges as a particularly informative biomarker in this context. Reduced SOCS3 expression in myeloid compartments has been associated with activation of Wnt/mTOR signaling, repression of autophagy, and enhanced survival of immunosuppressive eMDSCs (96). Thus, SOCS3-low signatures may identify tumors in which autophagy inhibition within myeloid cells contributes to immune evasion and tumor progression. Incorporating immune biomarkers alongside tumor cell–intrinsic metrics may therefore improve prediction of response to therapies targeting autophagy or upstream signaling pathways.

### 7.4 A three-dimensional stratification model: integrating Wnt, autophagy, and myeloid states

Building on these considerations, we propose a three-dimensional stratification framework integrating Wnt signaling activity, autophagy functionality, and myeloid immune status. In this model, tumors are categorized based on (i) Wnt/β-catenin activation level, (ii) autophagic flux competence and functional orientation, and (iii) degree of myeloid-driven immune suppression. Such an integrated approach acknowledges that therapeutic response is shaped by both tumor cell states and the surrounding immune ecosystem. For example, tumors with high Wnt activity, intact autophagic flux supporting survival, and prominent MDSC signatures may benefit from combined Wnt inhibition and autophagy blockade to simultaneously destabilize tumor cell programs and relieve immune suppression. In contrast, tumors with Wnt-driven oncogenic signaling but autophagy poised toward cytotoxic or pro-apoptotic outcomes may respond better to Wnt inhibition coupled with autophagy induction. This stratification framework emphasizes that precision modulation of autophagy requires coordinated evaluation of signaling, metabolism, and immunity, rather than reliance on a single biomarker axis. Importantly, this framework should not be interpreted as a universal model applicable to all breast cancer subtypes. Current evidence is strongest in Wnt-high, stem-like, therapy-resistant, and immune-suppressive tumor states, particularly in triple-negative breast cancer (TNBC), where Wnt/β-catenin activation, cancer stem cell enrichment, autophagy-dependent stress adaptation, and myeloid-driven immune suppression are frequently implicated. In contrast, the applicability of this model to estrogen receptor-positive (ER^+^) and HER2-positive (HER2^+^) breast cancers may be more context-dependent and may vary according to endocrine resistance, HER2-targeted therapy resistance, PI3K–AKT–mTOR activity, autophagic flux competence, and immune composition. Therefore, the proposed Wnt–mTOR–autophagy framework should be viewed as a subtype- and state-dependent model rather than a pan-breast cancer mechanism.

## 8 Challenges and future directions

Despite growing insights into the Wnt–mTOR–autophagy axis in breast cancer, several conceptual, technical, and translational challenges remain that must be addressed before these mechanisms can be fully leveraged for clinical benefit. Recognizing and resolving these limitations will be essential for advancing autophagy-targeted strategies in a rational and patient-specific manner.

### 8.1 Pitfalls in autophagy assessment: beyond static markers

A major challenge in the field lies in the accurate assessment of autophagy. Many studies rely heavily on static measurements such as LC3-II accumulation or p62/SQSTM1 abundance, which can be misleading when interpreted in isolation (82). Increased LC3-II levels may reflect enhanced autophagosome formation or, alternatively, impaired autophagosome clearance due to lysosomal dysfunction. Similarly, p62 accumulation often indicates defective autophagic flux rather than suppressed autophagy initiation. Failure to distinguish between these scenarios can lead to erroneous conclusions regarding whether autophagy is activated, inhibited, or functionally relevant. Future studies must therefore incorporate dynamic autophagy flux assays, including lysosomal inhibition with chloroquine or bafilomycin A1, as well as complementary readouts such as TFEB localization and lysosomal activity. Establishing standardized criteria for defining functional autophagy states will be critical for ensuring reproducibility and translational relevance.

### 8.2 Cell-type specificity of Wnt–autophagy crosstalk within the tumor ecosystem

Another underappreciated challenge is the pronounced cell-type specificity of Wnt–mTOR–autophagy interactions within the tumor ecosystem. While much of the existing literature focuses on tumor cell–intrinsic signaling, the same regulatory axis may exert distinct, and sometimes opposing, effects in immune cells, cancer-associated fibroblasts (CAFs), and endothelial cells. For example, autophagy may support survival and stemness in tumor cells while restraining differentiation or promoting apoptosis in myeloid populations. In CAFs, autophagy could influence extracellular matrix remodeling, metabolic coupling, and paracrine signaling, indirectly shaping tumor progression and immune infiltration (128–132). These divergent roles complicate therapeutic strategies aimed at globally modulating autophagy and highlight the risk of unintended consequences when targeting this pathway systemically. Dissecting cell-type–specific autophagy functions will require experimental models capable of resolving signaling and functional outcomes at high resolution. Conditional genetic models, lineage-specific reporters, and cell-type–restricted pharmacological interventions will be indispensable for defining how Wnt–autophagy crosstalk operates across different cellular compartments.

### 8.3 Therapeutic timing, sequencing, and toxicity of combination strategies

The clinical translation of Wnt–autophagy–targeted therapies also faces challenges related to treatment timing, sequencing, and toxicity. While preclinical studies support the efficacy of combining Wnt pathway inhibition with autophagy modulation, the optimal temporal configuration of such interventions remains unclear. Should Wnt signaling be inhibited prior to autophagy blockade to destabilize oncogenic programs, or should both pathways be targeted simultaneously to prevent compensatory survival responses? Moreover, systemic inhibition of autophagy—particularly through lysosomal inhibitors such as chloroquine—raises concerns regarding off-target effects and cumulative toxicity, given the essential role of autophagy in normal tissue homeostasis and immune function (133–135). Balancing therapeutic efficacy with tolerability will require careful dose optimization, treatment scheduling, and potentially the development of more selective autophagy modulators. Addressing these issues will necessitate rigorous evaluation of combination regimens in physiologically relevant models, including patient-derived organoids, syngeneic and humanized mouse models, and co-culture systems that recapitulate tumor–immune interactions.

### 8.4 Integrative technologies and future research directions

Advancing the field will depend on the integration of state-of-the-art technologies capable of resolving the spatial, temporal, and functional complexity of Wnt–mTOR–autophagy crosstalk. Single-cell and spatial transcriptomic approaches offer unprecedented opportunities to map pathway activity, autophagy states, and immune phenotypes across heterogeneous tumor landscapes (136, 137). When combined with spatial proteomics and metabolomics, these methods can illuminate how local microenvironmental cues shape autophagy-dependent cell states. Functional genomics approaches, including CRISPR-based loss- and gain-of-function screens, will be essential for identifying context-specific regulators of autophagy and for distinguishing causal drivers from correlative markers. Importantly, integrating autophagy modulation with immunotherapy models will allow evaluation of how reprogramming autophagy in tumor or immune cells influences antitumor immune responses and therapeutic synergy. Together, these approaches will facilitate a more precise and mechanistic understanding of when and how autophagy should be manipulated in breast cancer, paving the way for biomarker-driven and context-specific therapeutic strategies.

### 8.5 Therapeutic nuances of Wnt blockade and next-generation autophagy inhibition

Although Wnt inhibition provides a rational strategy for tumors with Wnt–β-catenin pathway dependency, therapeutic blockade of this pathway is not straightforward. Different intervention nodes may produce distinct biological outcomes. Upstream approaches, such as ligand neutralization, Frizzled blockade, or Porcupine inhibition, may be effective only in ligand-dependent tumors and may fail to suppress pathway activity when downstream alterations, β-catenin stabilization, pathway bypass, or compensatory RTK–PI3K–AKT–mTOR and MAPK–ERK activation are present (114). Therefore, Wnt blockade should not be treated as a single therapeutic category. Emerging strategies increasingly focus on downstream components, including β-catenin, β-catenin–TCF/LEF transcriptional complexes, coactivator interactions, and precision degrader approaches, although selectivity and toxicity remain major concerns because β-catenin also participates in normal tissue homeostasis and adherens junction integrity (138–140). Supportive evidence from glioblastoma models further illustrates this complexity. In a recent study, WNT974 combined with anti-PD-1 therapy improved antitumor immunity, but resistance was associated with compensatory activation of p-mTOR, p-ERK, and p-AKT signaling and remodeling of myeloid populations, including a shift from granulocytic MDSCs toward monocytic MDSCs (141). Although these findings were generated outside breast cancer, they highlight a general principle relevant to Wnt-targeted therapy: incomplete upstream Wnt blockade may permit adaptive signaling rewiring and immune-compartment compensation. Therefore, future breast cancer studies should evaluate Wnt inhibitors together with downstream pathway activity, mTOR status, autophagic flux, and myeloid composition rather than relying only on Wnt ligand or β-catenin expression. Importantly, no Wnt pathway inhibitor has yet been approved by the FDA for cancer therapy, underscoring the need for careful patient selection, biomarker-driven trial design, and combination strategies that anticipate compensatory pathway activation (139). For Wnt-driven breast cancers in which autophagic flux is retained and functions as a cytoprotective survival program, translational autophagy inhibition should also move beyond classical CQ or hydroxychloroquine. CQ and BafA1 are useful for flux assessment, but their clinical application is limited by potency, specificity, and toxicity concerns. More potent lysosome-directed autophagy inhibitors, including PPT1-targeting or dimeric chloroquine-like agents, may provide stronger inhibition of lysosomal function and autophagic flux. Lucanthone has been reported as an autophagy inhibitor with greater activity than chloroquine in breast cancer cell lines, and PPT1-targeting approaches have also been proposed to inhibit lysosomal function and tumor growth. Nevertheless, these agents should be discussed as investigational strategies rather than established standards, and their use in Wnt-driven breast cancer requires validation in subtype-specific, immune-competent, and flux-confirmed models. To facilitate translational interpretation, we summarized representative autophagy inhibitors and activators, their major target nodes, clinical or investigational status, and reported effects on Wnt/β-catenin signaling in **Supplementary Table 1**.

## 9 Conclusion

Accumulating evidence positions the Wnt/β-catenin–mTOR axis as a central, context-dependent gatekeeper of autophagy in breast cancer. Rather than acting as an isolated stress-response pathway, autophagy emerges as a signaling-integrated decision node whose functional orientation—cytoprotective versus cytotoxic—is dictated by upstream oncogenic and metabolic cues. Through coordinated regulation of autophagy initiation, lysosomal capacity, and flux, Wnt–mTOR signaling couples proliferative and stemness programs with stress adaptation, thereby shaping tumor cell survival, plasticity, and therapeutic resistance. Beyond tumor cell–intrinsic processes, this review highlights autophagy as a critical regulator of the tumor immune microenvironment. In particular, SOCS3 deficiency–driven activation of Wnt/mTOR signaling in early-stage myeloid-derived suppressor cells establishes autophagy repression as a mechanism sustaining immunosuppressive myeloid populations. This insight expands the functional relevance of autophagy from tumor metabolism to immune regulation and identifies myeloid autophagy as an emerging axis of immune escape in breast cancer. Targeting autophagy within immune compartments may therefore complement tumor-directed strategies and enhance antitumor immunity. Importantly, the apparent contradictions surrounding autophagy modulation in breast cancer can be reconciled through a context-specific framework. Whether autophagy should be induced or inhibited depends on the integrated state of Wnt signaling, mTOR activity, autophagic flux competence, and immune suppression. Therapeutic benefit is most likely to arise from rational combinations that align autophagy modulation with pathway dependency and cellular state, rather than from indiscriminate activation or blockade of autophagy. Looking forward, the clinical translation of Wnt–mTOR–autophagy–targeted strategies will hinge on biomarker-driven patient stratification and combination therapy design. Integrating signaling, autophagy, and immune biomarkers into multidimensional models, supported by single-cell and spatial profiling, will be essential for identifying patients most likely to benefit from tailored interventions. Ultimately, precision modulation of autophagy—guided by pathway context and tumor ecosystem dynamics—holds promise for improving therapeutic efficacy and overcoming resistance in breast cancer.
