# Supplementary material for: Wnt/β-Catenin–mTOR–autophagy crosstalk in breast cancer: context-dependent control of tumor progression, immune suppression, and therapeutic resistance
Source: Front Immunol. 2026 Jun 30;17:1842528. doi: 10.3389/fimmu.2026.1842528 (PMC13365330; doi:10.3389/fimmu.2026.1842528)
Supplement: Supplementary file 1 [file Table1.docx]

**Supplementary Table 1. Autophagy modulators and their reported effects on Wnt/β-catenin signaling.**

| **Autophagy modulator** | **Main autophagy-related action** | **Clinical/translational status** | **Effect on Wnt/β-catenin** | **Breast cancer relevance** | **References** |
| --- | --- | --- | --- | --- | --- |
| Chloroquine (CQ) | Lysosomal alkalinization; blocks autophagosome degradation and autophagic flux | Approved antimalarial; repurposed in cancer trials | May enhance antitumor effects when Wnt inhibition induces compensatory protective autophagy; also used to test whether Wnt-targeted therapy depends on autophagic flux | CQ sensitized TNBC cells to FZD7-targeted Wnt inhibition and targets TNBC cancer stem cells by inducing mitochondrial damage | DOI: 10.1016/j.canlet.2016.04.002 |
| Hydroxychloroquine (HCQ) | Lysosomal autophagy inhibitor; blocks late-stage autophagy | Approved antimalarial/autoimmune drug; widely tested in oncology combinations | Indirectly affects Wnt-associated survival programs by blocking protective autophagy; no strong evidence that HCQ directly inhibits canonical Wnt signaling | Useful as a clinically translatable autophagy inhibitor, but potency and specificity are limited | DOI: 10.1002/cncr.31335 |
| Bafilomycin A1 (BafA1) | Inhibits vacuolar H⁺-ATPase; blocks lysosomal acidification and autophagosome–lysosome degradation | Experimental tool; not clinically used | Useful for determining whether Wnt-associated changes reflect true autophagic flux or impaired degradation; not a therapeutic Wnt modulator | Mainly used in preclinical flux assays | DOI: 10.1002/cncr.31335, DOI: 10.4161/auto.5338 |
| Rapamycin / sirolimus | mTORC1 inhibition; induces autophagy initiation through ULK1 activation | Approved immunosuppressant; cancer use limited/indirect | Can suppress Wnt/β-catenin output in some tumor models, but the effect is context-dependent and may involve mTOR–autophagy feedback | Supports the concept that mTOR inhibition can link autophagy induction with altered Wnt signaling | DOI: 10.1007/s00432-020-03422-4, DOI: 10.1152/ajpcell.00368.2022 |
| Everolimus | mTORC1 inhibitor; promotes autophagy initiation | FDA-approved in several settings including HR⁺/HER2⁻ advanced breast cancer with endocrine therapy | May alter Wnt–mTOR–autophagy balance by inhibiting mTORC1, but direct evidence for Wnt suppression in breast cancer remains limited | Clinically relevant mTOR/autophagy-axis drug in breast cancer | DOI: 10.1056/NEJMoa1109653, DOI: 10.1007/s12325-013-0060-1, DOI: 10.1093/annonc/mdu456 |
| Metformin | AMPK activation and mTOR inhibition; can induce stress-associated autophagy | Approved antidiabetic drug; investigated in cancer prevention/therapy | Reported to inhibit Wnt/β-catenin signaling in some cancer contexts; may act through AMPK/mTOR and β-catenin regulation | Potential metabolic-autophagy modulator; breast cancer relevance is plausible but context-dependent | DOI: 10.1080/10715762.2018.1485075 |
| Resveratrol | Natural compound; induces autophagy in some contexts, partly through Wnt/β-catenin suppression and mTOR modulation | Nutraceutical/research compound; not standard cancer therapy | Inhibits Wnt/β-catenin signaling and induces autophagy, associated with reduced breast cancer stem-like traits | Directly relevant to BCSC/Wnt/autophagy discussion | DOI: 10.1371/journal.pone.0102535 |
| Lucanthone | Lysosome/autophagy inhibitor; disrupts autophagy more potently than CQ in some settings | Investigational/repurposed anticancer agent | Potentially blocks cytoprotective autophagy downstream of Wnt-active states; direct Wnt-specific evidence is limited | Reported to inhibit autophagy more effectively than CQ in breast cancer cell lines | DOI: 10.1074/jbc.M110.151324 |
| PPT1-targeting agents / dimeric CQ-like compounds | Lysosome-directed autophagy inhibition; PPT1 inhibition | Investigational | May provide stronger blockade of protective autophagy than CQ/HCQ; Wnt-specific effects require validation | Proposed for Wnt-driven tumors with retained protective autophagic flux | DOI: 10.1158/2159-8290.CD-18-0706 |
